# Calibration of the PROMIS Physical Function Item Bank in Dutch Patients with Rheumatoid Arthritis

**DOI:** 10.1371/journal.pone.0092367

**Published:** 2014-03-17

**Authors:** Martijn A. H. Oude Voshaar, Peter M. ten Klooster, Cees A. W. Glas, Harald E. Vonkeman, Erik Taal, Eswar Krishnan, Hein J. Bernelot. Moens, Maarten Boers, Caroline B. Terwee, Piet L. C. M. van Riel, Mart A. F. J. van de Laar

**Affiliations:** 1 Arthritis Center Twente and Psychology, Health & Technology, University of Twente, Enschede, The Netherlands; 2 Department of Research Methodology, Measurement and Data Analysis, University of Twente, Enschede, The Netherlands; 3 Department of Rheumatology and Clinical Immunology, Medisch Spectrum Twente, Enschede, The Netherlands; 4 Arthritis, Rheumatism, and Aging Medical Information System Program, Stanford University School of Medicine, Palo Alto, California, United States of America; 5 Department of Rheumatology and Clinical Immunology, Ziekenhuisgroep Twente, Almelo, The Netherlands; 6 Department of Epidemiology and Biostatistics, Vrije Universiteit Medical Center, Amsterdam, The Netherlands; 7 Department of Rheumatic Diseases, Radboud University Medical Centre, Nijmegen, The Netherlands; University of Michigan, United States of America

## Abstract

**Objective:**

To calibrate the Dutch-Flemish version of the PROMIS physical function (PF) item bank in patients with rheumatoid arthritis (RA) and to evaluate cross-cultural measurement equivalence with US general population and RA data.

**Methods:**

Data were collected from RA patients enrolled in the Dutch DREAM registry. An incomplete longitudinal anchored design was used where patients completed all 121 items of the item bank over the course of three waves of data collection. Item responses were fit to a generalized partial credit model adapted for longitudinal data and the item parameters were examined for differential item functioning (DIF) across country, age, and sex.

**Results:**

In total, 690 patients participated in the study at time point 1 (T2, N = 489; T3, N = 311). The item bank could be successfully fitted to a generalized partial credit model, with the number of misfitting items falling within acceptable limits. Seven items demonstrated DIF for sex, while 5 items showed DIF for age in the Dutch RA sample. Twenty-five (20%) items were flagged for cross-cultural DIF compared to the US general population. However, the impact of observed DIF on total physical function estimates was negligible.

**Discussion:**

The results of this study showed that the PROMIS PF item bank adequately fit a unidimensional IRT model which provides support for applications that require invariant estimates of physical function, such as computer adaptive testing and targeted short forms. More studies are needed to further investigate the cross-cultural applicability of the US-based PROMIS calibration and standardized metric.

## Introduction

Rheumatoid arthritis (RA) is one of the most prevalent rheumatic diseases, characterized by pain and swelling of the joints which may lead to significant disability. Patient-reported physical function is a core outcome domain in RA research [1], [2]. Physical function is typically assessed using standard, fixed-length questionnaires. Although often extensively validated, key limitations of these traditional questionnaires remain their static nature and limited measurement range and measurement precision, frequently leading to ceiling and floor effects and limited sensitivity to change [3]–[8]. Recent studies have suggested that these shortcomings may be overcome by item response theory (IRT) based item banking [9], [10]. IRT calibrated item banks can serve as a platform for tailored assessment of patient-reported outcomes, through developing targeted short forms or computerized adaptive tests (CATs). Both methods of assessment ensure that patients respond to questions that are more relevant to their specific level of disability and that only minimal questions need to be answered, while retaining or surpassing the measurement precision of fixed-length instruments.

The Patient-Reported Outcomes Measurement Information System (PROMIS) initiative has developed and calibrated item banks for assessing several important domains of health status, including physical function, across a wide variety of chronic diseases and conditions and the general population in the US [11]. Using data from the general population and several clinical samples in the US, all items in the item banks are calibrated on a common, standardized metric. Potentially, the PROMIS physical function (PF) item bank could also lead to improved assessment of physical function in clinical or comparative studies in RA. Indeed, recent studies have already shown a 20-item PROMIS PF short form to be more precise and more responsive to change than traditional questionnaires in RA [12]. Recently, the PROMIS PF item bank has been translated and culturally adapted for use among Dutch and Flemish populations. Pretesting of the translated items revealed that the items were understood by patients as intended and culturally appropriate for use in Dutch populations with arthritis [13], [14]. Before an item bank can be used in a new population, however, it should be demonstrated that data collected from that population can be fit to an appropriate IRT model. If this is the case, a latent metric specific for this population can be created that allows invariant estimates of the item parameters and physical function levels to be obtained (e.g., item parameters that are independent of the physical function level of the respondents used to calibrate the item bank) [15]. As a result, physical function estimates on a common scale may be obtained from any number and combination of items in the item bank and applications such as CATs and targeted short forms become possible. A second question that needs to be addressed is whether the relationship between observed physical function scores and the physical function trait measured by the item bank is equivalent to this relationship for the original population. If this is the case, this would provide evidence that the model parameters can be expressed on a common scale [16]. In case of the PROMIS PF item bank, this would mean that data from the specific population can be scored using the US-based PROMIS calibration and standardized metric, making scores directly comparable between populations.

The aims of the current study were to calibrate the Dutch-Flemish PROMIS PF item bank in a prospective cohort of Dutch patients with RA and to evaluate its measurement equivalence with data from the total PROMIS wave 1 calibration sample in the US and a smaller subset of US RA patients.

## Methods

### Patients

Data for this study were collected within the Dutch Rheumatoid Arthritis Monitoring (DREAM) registry. The DREAM registry is an observational multicenter cohort study that monitors the course of unselected RA patients in the Netherlands. Both patient-reported and clinical outcomes are collected and monitored using a web-based data acquisition and storage system. Patient-reported outcomes, including the Health Assessment Questionnaire disability index (HAQ-DI) and the SF-36 health survey, are completed preceding every visit to the outpatient clinic. Between September 2012 and September 2013, all participating patients from three DREAM hospitals were informed about the study and invited to participate upon logging on to their patient portals preceding their visit to the clinic.

### Data collection designs

#### Dutch DREAM data

**Figure 1:**
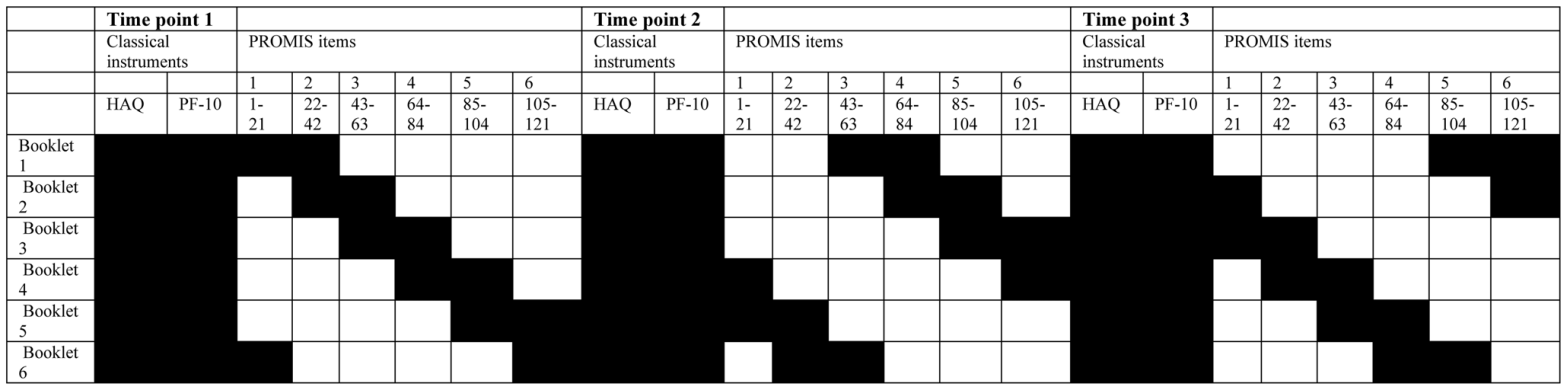
Sampling design of the Dutch calibration study.

To optimize data quality and minimize patient burden, an incomplete longitudinal design was used for calibrating the Dutch-Flemish item bank in the Dutch RA patients, in which different subsets of items (booklets) were administered to different patients. The booklets were linked using common items, making it possible to place all items on a single scale [17]. Since previous research has found that the number of common items within booklets improves the stability of IRT models estimated from incomplete calibration designs, [18] the item responses on the HAQ-DI and the SF-36 physical functioning scale (PF-10), the two most widely used measures of physical function in RA, were added to the calibration design. A graphical overview of the calibration design is presented in Figure 1.

Upon consenting to participate, patients were allocated randomly to one of six booklets. Besides the HAQ-DI and PF-10, each booklet contained two sets of approximately 20 of the 121 PROMIS PF items and each of the six sets featured in two booklets in such a way that half of the items in each booklet overlapped with the previous booklet and half with the next. On successive participations, patients were allocated to booklet N+2 (for N = 1,2,3,4) or N−4 (for N = 5,6), where N is the booklet that was administered at the preceding participation, so that patients completed the full item bank after three participations. The sample sizes of the six groups were approximately equal so that all items received an approximately equal number of responses.

From historical log data of physical function items in the DREAM registry, it was estimated that the majority of patients would need no more than 10 minutes to complete each booklet of approximately 40 items. An effort was made to balance the relative difficulty of the items in each booklet by ordering the items according to their peak statistical information on the latent IRT metric according to the US PROMIS wave 1 calibration results. As 20% of the PROMIS PF items has a different stem (i.e., ‘does your health now limit you…’ rather ‘than are you able to…’) and associated set of response options, each booklet contained a proportional number of these items.

#### US PROMIS wave 1 data

PROMIS wave 1 data for 14 candidate item pools, including three pools of physical function items, were collected between July 2006 to March 2007 from over 21,000 participants selected from both the US general population and specific clinical populations [19]. The data collection design of the wave 1 data consisted of both so-called ‘full bank administrations’, where participants were administered two sets of 56 items from only one or two item pools, and ‘block administrations’ where participants completed 14 blocks of seven items from all item pools. To avoid complicating the calibration design and analyses, we chose to model only the available full bank data from the general population sample and the block data available from the clinical sample of RA patients.

The full bank arm of the data collection design for physical function in the general population consisted of two booklets that were completed by two independent samples of 942 and 995 respondents, respectively. The booklets were complementary in that each PF item featured in only one booklet and together the booklets contained all 121 final items of the PROMIS PF item bank. Besides the PROMIS PF items, respondents completed the HAQ-DI or PF-10 or both. The HAQ-DI and PF-10 data were included in the calibrations in order to obtain a linked structure so that the US item parameters could be placed on a common latent scale, despite the lack of overlapping PROMIS PF items between the two booklets. Additionally, two clinical samples of 273 and 280 RA patients completed a booklet with a selection of seven items from each of the three PF item pools. Twenty-four of these 42 administered items were calibrated in the final US PROMIS PF item bank (13 and 11 items from each booklet, respectively).

### Measures

#### PROMIS physical function (PF) item bank

The PROMIS PF item bank measures self-reported, current capability to carry out activities that require physical actions, ranging from self-care (activities of daily living) to more complex activities that require a combination of skills, often within a social context. The final calibrated item bank contains 121 questions assessing the functioning of the upper extremities (dexterity), lower extremities (walking or mobility), and central regions (neck, back), as well as instrumental activities of daily living, such as running errands [19]. Each item is scored on a 5 point rating scale, with higher scores indicating better functioning. The Dutch-Flemish translation of the item bank was developed according to the universal PROMIS translation approach (http://www.nihpromis.org/measures/translations), which included extensive forward-back translation procedures, expert reviews, and cognitive debriefing interviews among Dutch and Flemish participants [20].

#### Health Assessment Questionnaire disability index (HAQ-DI)

The HAQ-DI contains 20 items measuring physical disabilities over the past week in eight categories of daily living: dressing and grooming, rising, eating, walking, hygiene, reach, grip, and activities [21]. Each item is scored on a 4-point rating scale from 0 (without any difficulty) to 3 (unable to do). Disability scores were calculated according to the alternative scoring rule, which does not account for the use of aids and help from others [22]. Category scores are averaged to produce a total score between 0 and 3, with higher values indicating more disability. The Dutch consensus version of the HAQ-DI was used in the DREAM data collection.

#### SF-36 Health Survey physical functioning scale (PF-10)

The PF-10 is one of the eight scales of the SF-36 Health Survey and consists of 10 items measuring perceived current limitations in a variety of physical activities on a 3-point response scale from 1 (yes, limited a lot) to 3 (no, not limited at all). Scores of the PF-10 items are summed and linearly transformed to range between 0 and 100, with higher scores indicating better physical functioning [23]. The Dutch version of the SF-36v2 was used in the DREAM study [24].

#### Additional patient-reported and clinical measures

The Dutch DREAM registry additionally collected patient-reported general health, disease activity, fatigue, and pain in the past week on 0–100 visual analog scales (VASs), with higher scores indicating worse status. Clinical data were collected during visits to the outpatient clinic, including a 28-tender joint count, 28-swollen joint count, and erythrocyte sedimentation rate. Together with the VAS general health, these measures were combined into a single index of clinical disease activity (DAS28) [25].

### Statistical analysis

All IRT analyses were performed with the MIRT software package [26]. The marginal maximum likelihood estimation procedure was utilized to estimate the model parameters and the latent physical function levels of patients were estimated using the expected a posteriori (EAP) method throughout all analyses. Latent physical function scores are expressed on a scale with a mean of 0 and SD of 1. A multidimensional generalization of the two-parameter generalized partial credit model (GPCM), suitable for the analysis of longitudinal, polytomous data [27], was used to model the Dutch data. In this model, the item parameters pertain to time point specific latent dimensions and the dependency between item responses at different time points is modeled by the correlation between the dimensions. The model allows patients' levels of physical function to change over time but item parameters are constrained to be equal across time points. To evaluate whether the item parameters were stable over time, the presence of longitudinal differential item functioning (DIF) was evaluated using regression analysis as proposed by Te Marvelde & Glas [28]. To this end, unidimensional GPCM estimates of the Dutch PROMIS data were obtained for each time point separately. The resulting threshold parameters were regressed on the threshold parameters emanating from one of the other two models in a series of univariate regression models. Individual items were considered to display statistically significant longitudinal DIF in case an item's 99% confidence interval did not intersect the regression line [28].

Fit of the longitudinal IRT model was assessed using Lagrange multiplier (LM) statistics, which evaluate whether observed item scores correspond to those expected by the item characteristic function [29]. To evaluate the magnitude of model violation of significant LM tests, effect size statistics (ES) were also obtained. These effect sizes are differences between average observed and expected scores across 3 total-score level groups. To compute these effect sizes, the patients were divided in 3 groups of approximately equal size obtaining low, intermediate, and high scores. The observed and expected scores were divided by the maximum attainable item score, such that a difference of, say, 0.10 indicated that the observed average score was 10% different from its expectation under the model. Items were considered to lack fit in case *P*'s<0.05 and ES statistics were >0.10 [30]. We first evaluated fit within time points by estimating the unidimensional GPCM 3 times, once for each time point. Subsequently, fit of the total multidimensional model, with item parameters constrained to be equal across time points and which includes the covariance matrix between time points, was evaluated. The Dutch data was evaluated for DIF across age (median split at 58 years) and sex. To this end the baseline model was extended by partitioning the booklets further according to age or sex and DIF was evaluated across two marginal distributions of physical function of males vs. females and younger vs. older patients, respectively. DIF across the marginal distributions was evaluated with an LM test for DIF [31].

Cross-cultural equivalence with the original US data was investigated first using the wave 1 general population data. The analysis was subsequently repeated on the independent subset of 25 items administered to the US RA patients [19]. US item parameters were obtained from a unidimensional GPCM and analysis of cross-cultural DIF was again performed with the regression analysis method outlined above [28]. To examine the impact of any observed DIF, US and Dutch baseline data were jointly modeled in a unidimensional GPCM with country-specific item parameters for those items flagged for cross-cultural DIF. The resulting EAP estimates were compared to those emanating from a model without country-specific item parameters. In both models, the mean was set to zero for US respondents (SD = 1). The agreement between the resulting latent EAP estimates was evaluated by calculating intraclass correlation coefficients (ICCs, model A,1) and the limits of agreement according to the Bland-Altman method [32]. Two independent data sets were available of US RA patients. The first sample (Stanford sample) contained 14 items administered to 273 patients and the second sample (Polimetrix sample) contained 10 items administered to 280 patients. To evaluate RA-related DIF, the baseline model of Dutch RA-patients was extended to incorporate these data. DIF was subsequently evaluated across three marginal distributions (Dutch, Stanford, and Polimetrix) using the LM test approach outlined above.

## Results

### Participant characteristics

Baseline data of 690 Dutch RA patients was available for analysis (Table 1). Of these, 489 and 311 patients completed booklets at T2 and T3, respectively. Average time between participations was 6.0 months (SD = 2.5) for T1 to T2 and 4. months (SD = 1.8) for T2 and T3. On average, Dutch patients had relatively low disease activity and high levels of physical function at baseline. Whereas the US general population and the combined RA samples had a balanced sex distribution, 64% of the Dutch RA patients were female, reflecting the greater prevalence of RA among women. The average level of physical function of US general population respondents was higher than that of Dutch RA patients according to the HAQ-DI and the average age of US general population respondents was lower.

**Table 1:** Sample characteristics.

	Dutch RA patients (N = 690)	US RA patients (N = 557)	US general population (N = 1937)
Female, N (%)	371 (63.6%)	293 (52.6%)	1004 (51.8)
Age, M (SD)	56.8 (11.8)	56.66 (10.9)	50.5 (18.3)
DAS28, M (SD)	2.1 (1.1)	-	-
VAS General health, M (SD)	25.1 (23.6)	-	-
VAS Disease activity, M (SD)	28.2 (23.4)	-	-
VAS Fatigue, M (SD)	33.1 (29.8)	-	-
VAS Pain, M (SD)	27.6 (22.7)	-	-
HAQ-ADI, M (SD)	0.5 (0.6)	-	0.2 (0.4)

VAS  =  Visual analog scale; DAS28 = 28-joint disease activity score.

### Evaluation of the longitudinal IRT model in the Dutch data

Table 2 presents an overview of the LM tests and the average observed and average expected item scores across three total score level groups for the PROMIS PF items administered in the odd booklets at T1 (see Figure 1). Results were similar for the even booklets and the other time points. The items are organized according to the point on the latent scale where they provide their optimum information, as an indication of the relative difficulty of the activities they refer to. As expected, more ‘easy’ items referred to simple activities of daily living, such as eating or getting up from a chair, while items involving increasingly higher levels of cardiopulmonary function were clustered around the higher end of the latent metric. For most items, average observed scores were quite high considering the 1–5 rating scale of the PROMIS items, reflecting the relatively high level of physical function of the sample. Item scores expected by the IRT model tended to be close to the observed item scores across total score groups, leading to an acceptable average ES of 0.01 for time point 1.

**Table 2:** Evaluation of item fit for PROMIS items in the odd booklets at time

Item code	Item stem	LM	P	ES	Score group					
					1		2		3	
					Obs	Exp	Obs	Exp	Obs	Exp
PFC53	Are you able to get in and out of bed?	0.53	0.77	0.02	3.91	3.86	4.54	4.50	4.86	4.95
PFB48	Does your health now limit you in taking a shower?	0.35	0.84	0.01	3.97	4.03	4.71	4.72	4.95	4.96
PFA15	Are you able to stand up from an armless straight chair?	1.13	0.57	0.01	3.88	3.77	4.76	4.72	5.00	4.99
PFC6	Are you able to walk a block (about 100 m) on flat ground? *	3.12	0.21	0.01	4.23	4.25	4.97	4.92	5.00	4.99
PFB26	Are you able to shampoo your hair?	0.14	0.93	<0.01	3.97	3.98	4.85	4.83	5.00	4.99
PFA45	Are you able to get out of bed into a chair?	1.26	0.53	0.01	4.40	4.32	4.80	4.85	5.00	4.99
PFA51	Are you able to sit on the edge of a bed?	0.01	0.99	<0.01	4.65	4.64	4.94	4.94	5.00	5.00
PFA30	Are you able to step up and down curbs?	1.46	0.48	0.01	4.13	4.24	4.84	4.89	5.00	4.99
PFC47	Are you able to be out of bed most of the day?	1.13	0.57	0.01	4.57	4.56	4.81	4.91	5.00	4.98
PFB18	Are you able to shave your face or apply makeup?	0.27	0.87	0.01	4.23	4.24	4.85	4.89	5.00	4.99
PFC46	Are you able to transfer from a bed to a chair and back?	2.85	0.24	0.02	4.28	4.14	4.84	4.90	5.00	4.99
PFA40	Are you able to turn a key in a lock?	2.34	0.31	0.02	3.83	3.98	4.71	4.64	4.97	4.97
PFB16	Are you able to press with your index finger (for example ringing a doorbell)?	2.41	0.30	0.01	4.40	4.40	4.92	4.84	5.00	4.99
PFC39	Are you able to stand without losing your balance for several minutes?	7.77	0.02	0.03	3.97	4.19	4.89	4.74	4.95	4.96
PFC51	Are you able to wipe yourself after using the toilet?	0.16	0.92	0.01	4.20	4.18	4.80	4.77	5.00	4.98
PFA54	Are you able to button your shirt?	0.27	0.88	0.01	3.86	3.79	4.59	4.60	4.89	4.91
PFB31	Are you able to open car doors?	2.00	0.37	0.02	4.19	4.03	4.65	4.67	4.98	4.97
PFB29	Are you able to lift a full cup or glass to your mouth?	1.93	0.38	0.01	4.28	4.35	4.93	4.87	5.00	4.99
PFA50	Are you able to brush your teeth?	1.79	0.41	0.01	4.32	4.36	4.93	4.88	5.00	4.99
PFC45	**Are you able to sit on and get up from the toilet?** †	2.88	0.24	0.01	3.98	4.02	4.90	4.83	5.00	4.99
PFB27	Are you able to tie a knot or a bow?	7.91	0.02	0.05	3.26	3.62	4.66	4.49	4.89	4.94
PFB20	Are you able to cut a piece of paper with scissors?	3.24	0.20	0.02	3.91	4.05	4.77	4.69	5.00	4.97
PFA44	Are you able to put on a shirt or blouse?	5.81	0.05	0.03	3.83	3.96	4.80	4.66	4.94	4.97
PFA35	Are you able to open and close a zipper?	3.76	0.15	0.02	3.88	3.99	4.89	4.80	5.00	4.99
PFB22	Are you able to hold a plate full of food?	1.34	0.51	0.01	4.03	4.03	4.66	4.79	4.97	4.97
PFA16	Are you able to dress yourself, including tying shoelaces and buttoning your clothes?	1.88	0.39	0.01	3.60	3.65	4.76	4.66	4.95	4.96
PFA38	Are you able to stand for short periods of time?	0.23	0.89	0.01	3.77	3.73	4.50	4.50	4.95	4.96
PFA48	Are you able to peel fruit?	1.96	0.38	0.02	3.69	3.75	4.65	4.53	4.93	4.92
PFB15	Are you able to change the bulb in a table lamp?	0.12	0.94	0.01	4.00	3.97	4.78	4.80	4.98	4.98
PFC49	**Are you able to water a house plant?** †	2.75	0.25	0.02	4.26	4.42	4.95	4.93	5.00	5.00
PFB33	Are you able to remove something from your back pocket?	2.95	0.23	0.03	3.83	4.05	4.83	4.78	4.97	4.98
PFB21	Are you able to pick up coins from a table top?	0.68	0.71	0.02	3.97	3.89	4.63	4.57	4.89	4.92
PFB10	Are you able to climb up five steps?	0.12	0.94	0.01	3.76	3.81	4.66	4.64	5.00	4.97
PFB19	Are you able to squeeze a new tube of toothpaste?	0.49	0.78	0.01	4.18	4.10	4.84	4.85	5.00	4.98
PFB36	Are you able to put on a pullover sweater?	0.73	0.70	0.01	4.17	4.09	4.76	4.80	4.95	4.96
PFC29	Are you able to walk up and down two steps?	0.97	0.61	0.02	3.89	3.77	4.59	4.71	4.92	4.91
PFA56	Are you able to get in and out of a car?	0.24	0.89	0.01	3.89	3.89	4.55	4.51	4.95	4.95
PFA36	**Are you able to put on and take off a coat or jacket?** †	1.51	0.47	0.02	3.74	3.83	4.58	4.50	4.98	4.95
PFA32	**Are you able to stand with your knees straight?**	2.44	0.30	0.02	4.20	4.10	4.90	4.84	5.00	4.99
PFA43	Are you able to write with a pen or pencil?	0.32	0.85	0.01	3.89	3.85	4.71	4.67	4.97	4.97
PFB49	Does your health now limit you in going for a short walk (less than 15 minutes)?	0.03	0.98	0.01	3.34	3.32	4.24	4.26	4.86	4.88
PFA22	Are you able to open previously opened jars?	1.09	0.58	0.01	3.78	3.68	4.49	4.50	4.88	4.88
PFB25	Are you able to push open a door after turning the knob?	1.65	0.44	0.02	4.42	4.32	4.82	4.87	5.00	4.98
PFB41	Are you able to trim your fingernails?	3.30	0.19	0.03	4.03	3.81	4.67	4.72	4.92	4.96
PFB23	Are you able to pour liquid from a bottle into a glass?	1.40	0.50	0.01	4.43	4.40	4.84	4.91	5.00	4.99
PFA52	Are you able to tie your shoelaces?	4.11	0.13	0.02	3.48	3.58	4.64	4.49	4.98	4.95
PFA49	Are you able to bend or twist your back? *	0.29	0.86	0.01	3.77	3.84	4.52	4.55	4.91	4.93
PFB3	**Does your health now limit you in putting a trash bag outside?** †	2.27	0.32	0.03	3.19	3.08	3.82	3.97	4.82	4.88
PFA9	Are you able to bend down and pick up clothing from the floor?	1.13	0.57	0.02	3.85	3.72	4.55	4.54	4.90	4.94
PFA37	Are you able to stand for short periods of time?	2.04	0.36	0.02	4.00	3.88	4.74	4.66	5.00	4.95
PFA17	Are you able to reach into a high cupboard?	1.98	0.37	0.02	3.31	3.24	4.34	4.22	4.89	4.88
PFB43	Does your health now limit you in taking care of your personal needs (dress, comb hair, toilet, eat, bathe)?	0.09	0.95	0.01	3.63	3.59	4.42	4.42	4.92	4.95
PFC31	Are you able to reach into a low cupboard?	0.64	0.73	0.01	3.43	3.44	4.50	4.39	4.92	4.91
PFB11	Are you able to wash dishes, pots, and utensils by hand while standing at a sink?	0.13	0.94	0.01	3.88	3.88	4.71	4.67	4.97	4.97
PFA18	Are you able to use a hammer to pound a nail?	0.10	0.95	0.01	3.45	3.43	4.58	4.60	4.92	4.94
PFB37	Are you able to turn faucets on and off?	0.67	0.72	0.01	3.80	3.78	4.78	4.73	4.92	4.96
PFB56	Are you able to lift one pound (0.5 kg) to shoulder level without bending your elbow?	0.47	0.79	0.01	3.47	3.47	4.68	4.62	4.92	4.93
PFC43	**Are you able to use your hands, such as for turning faucets, using kitchen gadgets, or sewing?** †	0.80	0.67	0.02	3.44	3.54	4.21	4.26	4.83	4.88
PFC52	Are you able to turn from side to side in bed?	2.25	0.32	0.02	3.45	3.59	4.63	4.55	4.90	4.95
PFB32	Are you able to stand unsupported for 10 minutes?	1.17	0.56	0.03	3.34	3.50	4.50	4.38	4.95	4.87
PFA53	Are you able to run errands and shop?	0.96	0.62	0.02	3.39	3.28	4.37	4.32	4.92	4.95
PFB17	Are you able to put on and take off your socks?	0.96	0.62	0.01	3.87	3.76	4.71	4.70	4.97	4.96
PFA28	Are you able to open a can with a hand can opener?	2.45	0.29	0.02	3.19	3.09	4.15	4.30	4.85	4.84
PFA21	Are you able to go up and down stairs at a normal pace? *	0.55	0.76	0.02	3.17	3.22	4.21	4.14	4.83	4.88
PFB13	**Are you able to carry a shopping bag or briefcase?** †	0.06	0.97	<0.01	3.30	3.31	4.12	4.14	4.91	4.90
PFA34	Are you able to wash your back?	2.38	0.30	0.02	3.15	3.13	4.37	4.22	4.80	4.85
PFC41	Are you able to sit down in and stand up from a low, soft couch?	3.48	0.18	0.03	2.89	3.11	4.06	3.93	4.73	4.70
PFC38	Are you able to walk at a normal speed?	0.09	0.95	0.01	3.23	3.17	4.31	4.31	4.91	4.91
PFB40	Are you able to stand up on tiptoes?	0.14	0.93	0.01	3.24	3.20	4.50	4.47	4.89	4.85
PFA25	Are you able to do yard work like raking leaves, weeding, or pushing a lawn mower? *	0.68	0.71	0.02	2.79	2.69	3.58	3.55	4.68	4.65
PFA29	**Are you able to pull heavy objects (10 pounds/ 5 kg) towards yourself?** †	0.46	0.79	0.01	2.74	2.74	3.74	3.68	4.77	4.70
PFA47	**Are you able to pull on trousers?** †	0.96	0.62	0.01	3.95	3.88	4.78	4.81	5.00	5.00
PFC56	Does your health now limit you in walking about the house?	2.21	0.33	0.02	3.54	3.78	4.56	4.54	4.95	4.96
PFA12	**Are you able to push open a heavy door?** †	0.59	0.75	0.01	2.97	2.89	4.03	4.06	4.79	4.79
PFA8	Are you able to move a chair from one room to another?	1.83	0.40	0.02	2.84	3.03	4.37	4.35	4.95	4.90
PFB54	Does your health now limit you in going OUTSIDE the home, for example to shop or visit a doctor's office?	0.16	0.92	0.01	3.73	3.76	4.79	4.77	5.00	4.97
PFC32	**Are you able to climb up 5 flights of stairs?** *	0.39	0.82	0.02	3.08	3.15	4.20	4.27	4.80	4.84
PFA42	Are you able to carry a laundry basket up a flight of stairs? *	4.66	0.10	0.04	2.74	2.71	3.70	3.99	4.91	4.81
PFB39	Are you able to reach and get down a 5 pound (2 kg) object from above your head?	2.72	0.26	0.04	2.34	2.59	3.57	3.62	4.51	4.65
PFA23	Are you able to go for a walk of at least 15 minutes?	0.52	0.77	0.02	3.41	3.28	4.37	4.36	4.92	4.90
PFA11	Are you able to do chores such as vacuuming or yard work?	0.57	0.75	0.02	2.94	2.88	3.71	3.79	4.83	4.70
PFB34	Are you able to change a light bulb overhead?	0.29	0.86	0.01	2.89	2.88	4.18	4.25	4.85	4.84
PFA6	Does your health now limit you in bathing or dressing yourself?	0.32	0.85	0.01	3.43	3.48	4.34	4.33	4.93	4.95
PFA55	**Are you able to wash and dry your body?** †	2.31	0.31	0.01	3.62	3.62	4.56	4.70	5.00	4.99
PFB14	Are you able to take a tub bath?	2.53	0.28	0.04	3.25	2.96	4.38	4.48	4.97	4.87
PFB12	Are you able to make a bed, including spreading and tucking in bed sheets?	2.42	0.30	0.03	2.69	2.84	4.03	4.17	4.92	4.82
PFA14	Are you able to carry a heavy object (over 10 pounds/5 kg)?	0.27	0.88	0.01	2.72	2.65	3.63	3.62	4.64	4.59
PFA31	Are you able to get up from the floor from lying on your back without help?	1.52	0.47	0.03	2.88	2.70	3.61	3.66	4.78	4.70
PFC40	Are you able to kneel on the floor?	1.60	0.45	0.03	2.54	2.50	3.37	3.62	4.60	4.56
PFB42	Are you able to stand unsupported for 30 minutes?	0.65	0.72	0.02	2.69	2.59	3.91	3.79	4.71	4.70
PFC37	Does your health now limit you in climbing one flight of stairs? *	0.49	0.78	0.01	3.18	3.25	4.35	4.40	4.90	4.93
PFB28	**Are you able to lift 10 pounds (5 kg) above your shoulder?**	6.43	0.04	0.05	2.68	2.26	3.59	3.69	4.69	4.65
PFC54	Does your health now limit you in getting in and out of the bathtub?	7.25	0.03	0.05	3.05	2.66	3.65	3.70	4.91	4.81
PFB8	Are you able to carry two bags filled with groceries 100 yards (100 m)?	3.58	0.17	0.04	2.15	2.05	3.43	3.08	4.44	4.41
PFA41	Are you able to squat and get up?	0.93	0.63	0.02	2.79	2.66	3.65	3.61	4.67	4.62
PFA10	Are you able to stand for one hour?	2.83	0.24	0.04	2.43	2.36	3.74	3.49	4.69	4.49
PFB9	Are you able to jump up and down?	0.58	0.75	0.02	2.62	2.51	4.16	4.10	4.85	4.80
PFA5	Does your health now limit you in lifting or carrying groceries?	1.22	0.54	0.02	2.45	2.55	3.43	3.32	4.51	4.53
PFA13	Are you able to exercise for an hour?	4.52	0.10	0.04	2.33	2.40	3.58	3.25	4.44	4.35
PFB24	Are you able to run a short distance, such as to catch a bus?	0.83	0.66	0.02	2.39	2.34	3.63	3.76	4.67	4.65
PFC10	Does your health now limit you in climbing several flights of stairs? *	3.02	0.22	0.04	3.22	2.96	4.14	4.19	4.95	4.84
PFC36	Does your health now limit you in walking more than a mile (1.6 km)?	0.76	0.68	0.02	2.77	2.77	4.27	4.14	4.73	4.81
PFB1	Does your health now limit you in doing moderate work around the house like vacuuming, sweeping floors or carrying in groceries?	2.15	0.34	0.03	2.35	2.42	3.43	3.25	4.50	4.57
PFB50	How much difficulty do you have doing your daily physical activities, because of your health?	0.51	0.78	0.01	2.74	2.76	3.59	3.52	4.56	4.59
PFB44	Does your health now limit you in doing moderate activities, such as moving a table, pushing a vacuum cleaner, bowling, or playing golf?	0.75	0.69	0.02	2.32	2.34	3.20	3.08	4.47	4.43
PFA33	Are you able to exercise hard for half an hour?	2.85	0.24	0.04	2.06	2.24	3.36	3.56	4.51	4.54
PFA3	Does your health now limit you in bending, kneeling, or stooping?	0.03	0.99	0.01	2.54	2.52	3.53	3.52	4.51	4.53
PFC13	Are you able to run 100 yards (100 m)?	4.63	0.10	0.04	1.89	1.89	3.08	3.52	4.50	4.55
PFB5	Does your health now limit you in hiking a couple of miles (3 km) on uneven surfaces, including hills? *	0.73	0.69	0.02	1.89	1.99	3.32	3.37	4.59	4.62
PFC12	Does your health now limit you in doing two hours of physical labor?	3.49	0.18	0.04	2.68	2.61	3.02	3.22	4.23	4.43
PFC35	Does your health now limit you in doing eight hours of physical labor?	4.17	0.12	0.04	1.33	1.52	2.50	2.62	4.03	4.15
PFA4	Does your health now limit you in doing heavy work around the house like scrubbing floors, or lifting or moving heavy furniture? *	0.11	0.95	0.01	1.59	1.58	2.47	2.51	4.23	4.13
PFB51	Does your health now limit you in participating in active sports such as swimming, tennis, or basketball?	3.41	0.18	0.05	2.23	2.01	2.45	2.64	4.02	3.85
PFA1	Does your health now limit you in doing vigorous activities, such as running, lifting heavy objects, participating in strenuous sports? *	1.48	0.48	0.04	1.56	1.44	1.95	2.05	3.69	3.47
PFB7	Does your health now limit you in doing strenuous activities such as backpacking, skiing, playing tennis, bicycling or jogging?	2.27	0.32	0.05	1.46	1.60	2.00	1.96	3.65	3.30
PFA19	Are you able to run or jog for two miles (3 km)?	2.12	0.35	0.02	1.29	1.26	1.25	1.40	2.59	2.57
PFA39	Are you able to run at a fast pace for two miles (3 km)?	3.27	0.19	0.03	1.25	1.35	1.45	1.60	2.51	2.60
PFC7	Are you able to run five miles (8 km)	2.16	0.34	0.03	1.16	1.14	1.23	1.38	2.67	2.49
PFC33	Are you able to run ten miles (16 km)?	99.00	1.00	0.02	1.09	1.07	1.00	1.06	1.59	1.46
PFB30	**Are you able to open a new milk carton?** †	1.07	0.58	0.02	2.85	3.01	3.97	3.93	4.71	4.75

LM  =  Lagrange multiplier statistic; ES =  Effect size statistic; Obs  =  Average observed item score; Exp  =  Average expected item score; Items flagged for cross-cultural DIF are presented in bold. *Activity is relatively more difficult to perform for the US general population sample; †Activity is relatively more easy to perform for US general population sample.

The number of items exhibiting lack of fit was very low for all three time points. For T1, T2 and T3 respectively, only 14 (3%), 12 (3%) and 5 (1%) items demonstrated misfit according to the LM test. Moreover, ESs exceeded 0.10 only for two items, both at T3 (PFA9, ES = 0.10 and PFA15, ES = 0.11) The item parameters were stable over time, with all correlations between threshold parameters at different time points exceeding 0.90 and all of the 99% confidence intervals intersecting the regression line in the three univariate regression analyses.

In the subsequent evaluation of the longitudinal (multidimensional) model, 6.3% of item level fit statistics showed lack of fit to the model, which corresponds approximately to the level of significant item tests expected based on chance. None of the items showed lack of fit in both or, in case of the HAQ-DI and PF-10 items, all booklets that it was included in, nor did any item show misfit across time points. The multidimensional IRT model provides estimates of the correlation of PF over the three different time points. The correlation between between latent PF levels across the three time points ranged from 0.73 between T1 and T3 to 0.87 between T1 and T2, indicating that physical function levels were quite stable over time. The overall conclusion was that model fit was acceptable.

### DIF across age and gender

Seven items demonstrated DIF for sex, while five items showed DIF for age in the Dutch RA sample at baseline (Table 3). For all items flagged for sex DIF, men reported slightly higher scores than expected by the IRT model, whereas women reported lower scores than expected, indicating that the activities were easier for male RA patients. Likewise, all items flagged for age DIF, except item PFA53 (‘Are you able to run errands and shop?’) were more easily endorsed by younger rather than older patients.

**Table 3:** Differential item functioning (DIF) across age and sex in the Dutch RA sample.

Item code	Item stem	LM	P	ES	Obs	Exp	Obs	Exp
Sex					Female		Male	
PFB28	Are you able to lift 10 pounds (5 kg) above your shoulder?	13.60	<0.01	0.31	3.10	3.39	4.09	3.74
PFA12	Are you able to push open a heavy door?	8.38	<0.01	0.19	3.60	3.76	4.29	4.06
PFA14	Are you able to carry a heavy object (over 10 pounds/5 kg)?	9.56	<0.01	0.28	3.37	3.56	4.17	3.81
PFB39	Are you able to reach and get down a 5 pound (2 kg) object from above your head?	5.51	0.02	0.20	3.45	3.64	4.07	3.84
PFB14	Are you able to take a tub bath?	4.53	0.03	0.16	3.89	4.08	4.35	4.22
PFC45	Are you able to sit on and get up from the toilet?	5.00	0.03	0.10	4.64	4.56	4.61	4.72
PFA29	Are you able to pull heavy objects (10 pounds/ 5 kg) towards yourself?	4.32	0.04	0.18	3.54	3.66	4.16	3.92
					Older		Younger	
PFC13	Are you able to run 100 yards (100 m)?	10.42	<0.01	0.25	2.93	3.27	3.64	3.45
PFC35	Does your health now limit you in doing eight hours of physical labor?	6.32	0.01	0.22	2.37	2.60	3.01	2.79
PFC12	Does your health now limit you in doing two hours of physical labor?	5.07	0.02	0.14	3.25	3.42	3.62	3.50
PFA53	Are you able to run errands and shop?	5.84	0.02	0.11	4.30	4.16	4.21	4.29
PFA4	Does your health now limit you in doing heavy work around the house like scrubbing floors, or lifting or moving heavy furniture?	4.45	0.03	0.14	2.46	2.60	3.01	2.87

LM  =  Lagrange multiplier statistic; ES =  Effect size statistic; Obs  =  Average observed item score; Exp  =  Average expected item score.

### Equivalence with PROMIS wave 1 data

**Figure 2:**
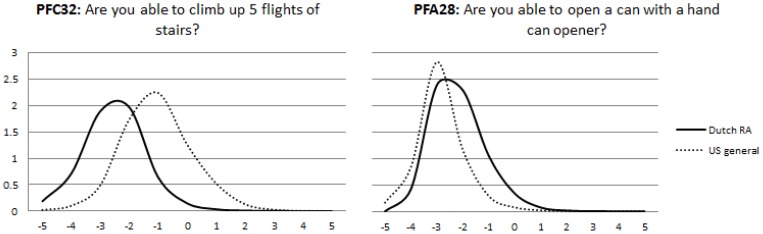
Country specific local measurement precision of two culturally biased items.

To evaluate measurement equivalence, US item parameters were obtained and compared with the Dutch item parameters using the regression analysis approach. Twenty-five items showed at least some level of uniform DIF in the regression analysis. For 11 of these items, Dutch patients were more likely to endorse lower response options according to the item response curves, indicating that these activities were relatively more difficult for them compared to the US general population. All these items involved the use of the hand or arms (see Table 2). Twelve items were more difficult for US respondents, of which five involved climbing stairs. Consequently, all items referring to climbing stairs were more precise at lower levels of overall physical function in the Dutch RA patients, whereas items involving dexterity tended to have better measurement precision at higher levels of function, as illustrated by two typical item information curves in Figure 2.

In the analysis of cross-cultural DIF in Dutch and US RA patients, the mean was set to zero for the Polimetrix sample and the latent means of the Dutch and Stanford sample were respectively −0.07 and 0.09, indicating that physical function levels were comparable between the samples. Seven out of 24 items showed significant DIF in the analysis (table 4).

**Table 4:** Differential item functioning (DIF) across Dutch and US RA patients.

		LM	P	ES	OBS	EXP	OBS	EXP
					Dutch		US	
PFB27	Are you able to tie a knot or a bow?	7.49	0.01	0.11	4.37	4.49	4.72	4.62
PFB20	Are you able to cut a piece of paper with scissors?	2.55	0.11	0.05	4.58	4.63	4.83	4.78
PFA17	Are you able to reach into a high cupboard?	1.07	0.30	0.04	4.13	4.17	4.49	4.46
PFB16	Are you able to press with your index finger (for example ringing a doorbell)?	0.30	0.58	0.02	4.75	4.76	4.89	4.87
PFA19	Are you able to run or jog for two miles (3 km)?	3.67	0.06	0.07	1.74	1.85	2.03	2.01
PFB21	Are you able to pick up coins from a table top?	0.41	0.52	0.03	4.49	4.52	4.73	4.71
PFA23	Are you able to go for a walk of at least 15 minutes?	12.71	<0.01	0.19	4.21	3.98	4.05	4.20
PFA22	Are you able to open previously opened jars?	5.39	0.02	0.09	4.35	4.44	4.56	4.48
PFB25	Are you able to push open a door after turning the knob?	<0.01	0.95	<0.01	4.71	4.71	4.75	4.74
PFB17	Are you able to put on and take off your socks?	0.36	0.55	0.02	4.45	4.47	4.62	4.60
PFB19	Are you able to squeeze a new tube of toothpaste?	4.41	0.04	0.07	4.63	4.70	4.86	4.80
PFB22	Are you able to hold a plate full of food?	1.32	0.25	0.04	4.57	4.61	4.69	4.65
PFA28	Are you able to open a can with a hand can opener?	7.23	0.01	0.13	4.05	4.19	4.35	4.24
PFB24	Are you able to run a short distance, such as to catch a bus?	4.97	0.03	0.13	3.53	3.40	3.30	3.42
PFA18	Are you able to use a hammer to pound a nail?	2.04	0.15	0.06	4.28	4.35	4.58	4.53
PFA16	Are you able to dress yourself, including tying shoelaces and buttoning your clothes?	1.49	0.22	0.03	4.41	4.45	4.59	4.56
PFA20	Are you able to cut your food using eating utensils?	8.65	<0.01	0.10	4.51	4.62	4.81	4.73
PFB23	Are you able to pour liquid from a bottle into a glass?	0.06	0.80	0.01	4.74	4.74	4.75	4.76
PFA21	Are you able to go up and down stairs at a normal pace?	30.32	<0.01	0.24	4.12	3.87	3.89	4.12
PFA25	Are you able to do yard work like raking leaves, weeding, or pushing a lawn mower?	7.77	0.01	0.12	3.67	3.55	3.53	3.64
PFB26	Are you able to shampoo your hair?	1.89	0.17	0.04	4.64	4.68	4.78	4.75
PFA15	Are you able to stand up from an armless straight chair?	35.86	<0.01	0.19	4.51	4.31	4.32	4.50
PFB15	Are you able to change the bulb in a table lamp?	1.44	0.23	0.04	4.62	4.66	4.83	4.79
PFB18	Are you able to shave your face or apply makeup?	0.95	0.33	0.03	4.72	4.75	4.88	4.85

### Impact of cross-cultural DIF

In the joint calibration of the Dutch RA data and the US general population data, with country-specific item parameters for the 25 DIF items, the mean of the latent physical function scores was set to 0 (SD = 1) for the US sample and the mean for Dutch RA patients was −1.18 (SD = 1.21), illustrating the considerably lower level of physical function of the Dutch RA patients. This estimate was very close to that observed in the original model without country-specific item parameters (M = −1.01, SD = 1.08), suggesting that the observed item DIF had little impact influence on the average total estimate obtained from all administered items. Moreover, agreement between total estimates was high (ICC = 0.99) and the limits of agreement were narrow, ranging from −0.23 to 0.25 in the Dutch data and from −0.20 to 0.18 in the US data.

## Discussion

This study presents the preliminary calibration and cross-cultural evaluation of the Dutch-Flemish translation of the PROMIS physical function (PF) item bank for Dutch patients with RA. The findings of the study indicate that the PROMIS PF item bank is a promising tool for applications such as CAT and tailored short forms in RA patients. However, some concerns remain regarding its cross-cultural measurement equivalence. Using the US-based standardized PROMIS calibration and metric requires further study.

The first principal finding of the current study was that the item bank could be successfully calibrated in a sample of Dutch patients with RA using an appropriate IRT model. To our knowledge, this is the first study to actually demonstrate that the full PROMIS PF item bank can be fitted to an appropriate IRT model in an RA sample. Therefore the current study provides support for the validity of applications of the item bank that require invariant estimates of the item and person parameters, such as CAT or short forms using a metric specific to Dutch patients with RA.

As a general rule, the stability of item parameters increases with more data. In that sense, the item parameters obtained in the current study should be considered preliminary and data that will be collected in future studies with the item bank in Dutch RA patients can be used to update the calibrations. Several ongoing studies in the Netherlands are evaluating the item bank in other patient groups. Future studies should evaluate the equivalence of the resulting item parameters across conditions to evaluate whether a common Dutch metric can be created.

The second principal finding of the study was that 25 of the PROMIS items (20%) showed substantial cross-cultural uniform DIF. The relatively high number of DIF items was not unexpected given that many items assess similar content (e.g. climbing stairs). Moreover, similar percentages of items with cross-cultural DIF are generally identified in scales with fewer items [30], [33]. Interestingly, all the PROMIS physical function item bank items that involve climbing stairs were more difficult for the US general population sample, compared to Dutch RA patients. This replicates findings in an earlier study we performed on the cross-cultural equivalence of HAQ-II in US and Dutch RA patients [30]. One speculative explanation for this repeated finding could be that Europeans are more accustomed to climbing stairs, since stairs are more prevalent in Europe, both in domestic and communal settings. However the US and Dutch sample might have also differed on key variables that might explain the observed DIF. For example body mass index has been linked to stair climbing in previous studies [34]. It would be interesting for future studies to evaluate the presence of body mass index related DIF in the PROMIS physical function items. By contrast, most items that were found to be more difficult for Dutch RA patients refer to activities involving the hands or the arms. This was not a surprising result, considering that disability of particularly the hands is a well-known clinical feature in RA. In fact, we had anticipated to find more DIF items between RA and the general population sample for items measuring dexterity. However, it should be noted that DREAM registry includes patients upon diagnosis with very early RA and these patients are treated aggressively. This is reflected in the average level of disease activity being below the commonly used DAS28 remission criterion of 2.6 and the low levels of disability observed, compared with international benchmarks in RA [35], [36]. Therefore, typical manifestations of RA-related disability may have been absent for many patients in the current study. Moreover, all items with collapsed response options involved measuring disability of the hands and these items showed severe distributional problems, even in the Dutch RA data with very few patients endorsing the lower response options. These two factors limit the sensitivity of the analyses with respect to RA-related DIF, and therefore studies in RA populations with more pronounced disease are desirable.

The results of the DIF analyses suggest that the Dutch RA data is not strictly equivalent to US general population data at the level of individual items, which was also observed in a previous study evaluating a Spanish language version of the item bank [37]. A limitation of the study design is that it cannot be definitively concluded whether observed differences in response probabilities conditional on overall level of function occurred because of disease characteristics or cross-cultural differences, since not all items were administered to US RA patients and no general population Dutch data is yet available. However, previous studies have generally shown European versions of physical function instruments to be equivalent to US versions in arthritis populations [30], [33], while substantial DIF has been observed across rheumatic conditions in one previous study [38]. It also seems unlikely that observed DIF occurred as a result of translation errors, given the rigorous approach in translating and that all items refer to everyday activities that are very common in both US and The Netherlands. For these reasons, more studies are needed before firm conclusions regarding the measurement equivalence can be made. If such studies consistently identify certain items to exhibit DIF, their item parameters can still be expressed on a common metric by assigning group-specific item parameters to biased items. This allows cross-cultural comparison even in the presence of significantly biased items and physical function levels to be expressed on the PROMIS standardized metric if this is desired. In the mean time we recommend that those interested in expressing physical function levels of Dutch RA patients on the PROMIS standardized metric to select only items that were not flagged for DIF in the current study.

In the analysis of impact of DIF on total EAP estimates of physical function, we observed that biased items appeared to have a negligible influence on total physical function estimates from all items that were administered to patients at baseline. It should be stressed though that patients were administered between 48 and 72 items which is likely to be greater than the number of items that will be administered in practical applications of the item bank. In a recent validation study of a PROMIS PF CAT only four items were administered on average to obtain physical function estimates [39]. The impact of DIF on physical function estimates is likely to be greater in such situations, provided that the item characteristics of biased items make them likely to be selected in such an application. Future studies should further evaluate the impact on physical function estimates in situations were fewer items are administered.

In the current study we used different methods to identify DIF. Whenever possible, DIF was evaluated using LM statistics. An advantage of this method is that violations of model assumptions can be investigated within a framework that directly pertains to the observed scores. As a result, the magnitude and direction of DIF can also be directly inferred from a weighted difference between average observed and average expected scores. In the regression analysis the direction of DIF had to be inferred indirectly by inspecting the response curves and item information functions visually. A limitation of the DIF analysis is therefore that no qualifications regarding the magnitude of DIF could be given in the current study of equivalence with the PROMIS wave 1 general population data. The reason we resorted to the regression analysis in the analysis of cross-cultural equivalence was that the US general population data suffered from severe ceiling effects, with the majority of respondents endorsing the higher response options. Consequently, insufficient variability was present within total score level groups for the LM test to produce interpretable results. For this reason also, no indication of model fit could be given for the US data. The longitudinal DIF analysis could not be performed with the LM test since the test compares scores on individual items between two groups, but in the longitudinal design, each item was presented to each patient only once.

In summary, the results of this study show that the PROMIS physical function item bank could be fitted to an IRT model that assumes physical function to be a unidimensional trait. However, a substantial number of its items showed statistically significant DIF compared to the US general population wave 1 data. Although the impact of observed DIF on physical function estimates was minimal in this study, more studies are needed to evaluate the validity of the PROMIS standardized metric in RA patients in the Netherlands.
